# Synergic Crosstalk between Inflammation, Oxidative Stress, and Genomic Alterations in BCR–ABL-Negative Myeloproliferative Neoplasm

**DOI:** 10.3390/antiox9111037

**Published:** 2020-10-23

**Authors:** Alessandro Allegra, Giovanni Pioggia, Alessandro Tonacci, Marco Casciaro, Caterina Musolino, Sebastiano Gangemi

**Affiliations:** 1 Department of Human Pathology in Adulthood and Childhood “Gaetano Barresi”, Division of Hematology, University of Messina, 98125 Messina, Italy; cmusolino@unime.it; 2 Institute for Biomedical Research and Innovation (IRIB), National Research Council of Italy (CNR), 98164 Messina, Italy; giovanni.pioggia@cnr.it; 3 Clinical Physiology Institute, National Research Council of Italy (IFC-CNR), 56124 Pisa, Italy; atonacci@ifc.cnr.it; 4 School and Operative Unit of Allergy and Clinical Immunology, Department of Clinical and Experimental Medicine, University of Messina, 98125 Messina, Italy; mcasciaro@unime.it (M.C.); gangemis@unime.it (S.G.)

**Keywords:** myeloproliferative neoplasms, essential thrombocythemia, polycythemia vera, myelofibrosis, inflammation, oxidative stress, ROS, driver mutation, thrombotic complication

## Abstract

Philadelphia-negative chronic myeloproliferative neoplasms (MPNs) have recently been revealed to be related to chronic inflammation, oxidative stress, and the accumulation of reactive oxygen species. It has been proposed that MPNs represent a human inflammation model for tumor advancement, in which long-lasting inflammation serves as the driving element from early tumor stage (over polycythemia vera) to the later myelofibrotic cancer stage. It has been theorized that the starting event for acquired stem cell alteration may occur after a chronic inflammation stimulus with consequent myelopoietic drive, producing a genetic stem cell insult. When this occurs, the clone itself constantly produces inflammatory components in the bone marrow; these elements further cause clonal expansion. In *BCR*–*ABL1*-negative MPNs, the driver mutations include *JAK 2*, *MPL*, and *CALR*. Transcriptomic studies of hematopoietic stem cells from subjects with driver mutations have demonstrated the upregulation of inflammation-related genes capable of provoking the development of an inflammatory state. The possibility of acting on the inflammatory state as a therapeutic approach in MPNs appears promising, in which an intervention operating on the pathways that control the synthesis of cytokines and oxidative stress could be effective in reducing the possibility of leukemic progression and onset of complications.

## 1. Introduction

### 1.1. Myeloproliferative Neoplasms and Inflammation

BCR–ABL-negative myeloproliferative neoplasms (MPNs) are an assorted set of acquired clonal diseases in which an anomalous hematopoietic stem cell alters myeloid progenitors, causing the increased generation of one or more forms of myeloid cells. The most frequent MPNs are polycythemia vera (PV), essential thrombocythemia (ET), and myelofibrosis (MF) [1,2].

In recent years, several findings have suggested that chronic inflammation may play an essential role in the onset and progression of MPNs [3,4,5,6].

It has been theorized that the starting event for this acquired stem cell alteration might be the consequence of a long-lasting inflammatory stimulus with subsequent chronic myelopoietic drive, finally provoking genetic stem cell damage. The persistent and self-maintaining generation of inflammatory products from in vivo stimulated progenitors and leukocytes causes a condition of chronic oxidative stress with augmented concentrations of reactive oxygen species (ROS) in the bone marrow (BM), which, in turn, are able to provoke the generation of a high-risk microenvironment. When this insult happens, the clone itself constantly produces inflammatory components in the BM. These elements further increase clonal expansion, thus leading to a positive feedback loop. A relationship between oxidative damage and the onset of leukemic diseases has, in fact, previously been observed [7,8,9,10].

It has been proposed that MPNs represent a human inflammation archetype for tumor progress, with chronic inflammation being the driving element of the transition from essential thrombocythemia (ET) over PV to the later myelofibrotic tumor phase [11]. In an animal experimental model, inflammatory components were shown to contribute to modification of the BM milieu into a leukemic niche that blocks regular hematopoiesis and supports leukemic stem cell development and myelofibrosis [12,13].

Thus, high sensitivity C-reactive protein is increased in numerous subjects with MPNs [14], and concentrations of circulating pro-inflammatory cytokines are increased in most subjects, being highly disproportionate in advanced MF [15,16,17].

However, transcriptional alterations present in patients with MPNs can contribute to the genesis of an inflammatory state. Entire blood gene expression analyses have reported inflammation and immune genes to be significantly deregulated [18,19]. Wong et al. investigated the expression of inflammatory genes in BM biopsies of MPN subjects. The authors recognized gene expression configurations that differentiate pre-fibrotic MPNs from fibrotic MPNs. Moreover, pre-fibrotic and fibrotic MPNs can be divided into two forms, with different behaviors in pathways correlated to inflammation, including cytokines, chemokines, and interferon response [20].

The previous claims have been confirmed by recent studies that emphasize the relationship between chronic Philadelphia-negative (Ph−) myeloproliferative diseases and the inflammasome. The word inflammasome is employed to define a high molecular weight cytosolic complex in activated immune cells. The innate immune response is mediated by inflammasomes; they are composed of large multimeric intracellular complexes that are capable of regulating stimulation of the proteolytic enzyme caspase-1 which, in turn, controls the proteolytic maturation of cytokines and regulates an inflammatory type of cell death, called pyroptosis, by cleavage of gasdermin D. Inflammasome stimulation causes an inflammatory response, resulting in either anti- and/or pro-tumor proliferation. Pattern recognition receptor (PRR), an apoptosis-associated speck-like protein, and the cysteine protease caspase-1 are the general components of inflammasomes. Several different families belonging to PRRs have been described, including Toll-like receptors (TLRs), nucleotide-binding oligomerization domain (NOD)-like receptors (NLRs), Rig-I-like receptors (RLRs), absent in melanoma 2 (AIM2)-like receptors (ALRs), and C-type lectin receptors (CLRs) [21].

Furthermore, some genes usually involved in inflammasome activation have been shown to be significantly overexpressed in MPNs [22].

### 1.2. Inflammatory Cytokines and MPNs

As reported above, increased concentrations of several cytokines and chemokines have been discovered in MPN subjects compared to healthy controls [15]. The detrimental roles of these compounds are also sustained by the finding that JAK2 inhibition can reduce the appearance of MPN traits in vivo through the reduction of cytokine delivery in mutant and non-mutant cells [23]. Furthermore, it has been claimed that precise targeting of some of the cytokines modified in MPNs may have better clinical results than those achieved with JAK2 inhibitors [23].

**Figure 1:**
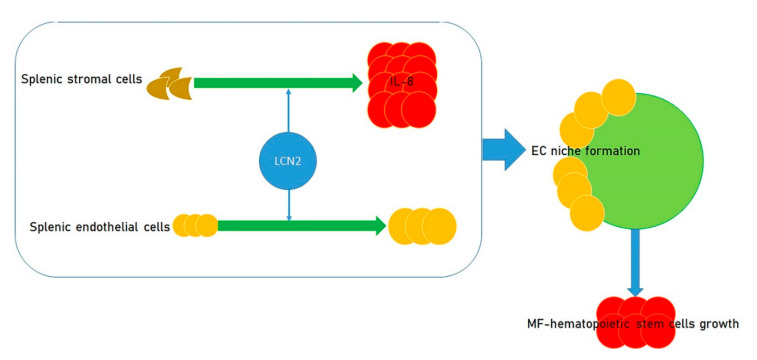
Effect of LCN2 on the growth of myelofibrosis (MF) hematopoietic stem cells. LCN2 fosters the growth of splenic endothelial cells, causing the enhanced production of IL-8 by splenic stromal cells. Such an increase plays a role in the formation of an endothelial cell niche, which, in turn, promotes the growth of myelofibrosis hematopoietic stem cells.

A modified cytokine pathway that has possible clinical relevance in MF is the lipocalin-2 (LCN2)/interleukin-8 (IL-8) axis and its downstream signaling through NF-κB. There is some indication that the events provoking alterations in the milieu in MF BM and spleen are due, at least in part, to LCN2 [24]. LCN2 increased the growth of splenic endothelial cells and caused increased production of IL-8 by splenic stromal cells. This increase contributes to the formation of an endothelial cell niche favoring the growth of MF hematopoietic stem cells [25,26] (Figure 1). This endothelial cell niche can be altered in vitro by reparixin, an antagonist of the receptors for IL-8 CXCR1/2 [27,28], which are present in high abundance on MF spleen CD34+ cells. A clinical trial to evaluate the actions of reparixin in MF has been proposed by the Myeloproliferative Neoplasm Research Consortium.

It has been established that the concentrations of other cytokines and chemokines differ between the three MPN diseases; for instance, the plasma of MF subjects showed greater amounts of IFN-gamma, IL-17A, and IL-12p70 than that of ET subjects and greater plasma concentrations of TNF-alpha, IL12-p70, IL-4, and GM-CSF than PV subjects. ET subjects presented greater plasma concentrations of RANTES than control and PV subjects, as well as greater concentrations of macrophage inflammatory protein (MIP-1) than PV subjects (Table 1). IL-12 and IL-17 are pro-inflammatory cytokines, whose modified concentrations have been linked to the physiopathology of several types of tumors [29,30]. RANTES, a chemokine correlated to tumor expansion, acts as a growth factor, promotes angiogenesis, and contributes to immune evasion mechanisms [31,32].

Øbro et al. presented a study of serum cytokine levels in more than 400 MPN subjects and identified an ET-specific inflammatory cytokine profile consisting of eotaxin, GRO-α, and Epidermal Growth Factor (EGF). Concentrations of two of these substances (GRO-α and EGF) in ET subjects have been correlated with disease transformation in initial sample collection (GRO-α) or longitudinal sampling (EGF). Furthermore, CD56^+^CD14^+^ pro-inflammatory monocytes have been recognized as a new source of augmented GRO-α concentrations [33]. This study highlights the relevant role of chronic inflammation for MPN disease progression and how tools (e.g., the evaluation of circulating inflammatory cytokines) might complete genomic profiling by evaluating prognosis and checking disease transformation to myelofibrosis [34].

The concentrations of IL-4 and IL-10, two anti-inflammatory cytokines, were also increased in all MPN subjects, indicating a general effort to counterbalance the disproportionate pro-inflammatory response. Moreover, in MPN, hematopoietic cells have shown an intensified response to inflammatory cytokines and growth factors such as IL-3, erythropoietin, granulocyte-macrophage colony-stimulating factor, and insulin-like growth factor-1 [35].

Autocrine stimulus of cytokine production can occur as a result of either malfunctioning cytokine receptors or the constitutive stimulation of several signaling pathways. Moreover, clonal evolution has been correlated to genomic instability, as generated by the effects of inflammatory cytokines on transcription elements such as NF-κB and STAT3 [36,37].

Generally, the transcription factor NF-κB is activated in reaction to infectious agents and pro-inflammatory cytokines, involving the modified expression of several genes and, in turn, is capable of supporting tumorigenesis [38]. STAT3 is also stimulated by numerous inflammatory cytokines, including IL-6, and growth factors such as epidermal growth factor [39,40]. Thus, STAT3 is triggered during inflammation and is activated in numerous tumors [41,42].

The increase in inflammatory cytokines found in MPNs not only influences the onset of the disease but also seems to be able to influence their evolution and prognosis. Vaidya et al. reported that the increased plasma concentrations of 13 cytokines were correlated with a lower overall survival in a group of PV and MF subjects [43]. Moreover, Tefferi et al. demonstrated increased concentrations of monokines and cytokines such as MCP-1 and vascular endothelial growth factor, while concentrations of IL-2R, IL-8, IL-12, and IL-15 were independently prognostic in primary myelofibrosis, where the amount of cytokine was linked with shorter survival in MF subjects [15].

In other studies on MF subjects, the correlation of specific cytokines with lower survival (IL2R, IL8), transfusion dependence (IL2R, IL8), higher number of leukocytes (IL2R), circulating blasts (IL-8), lower platelet count (IP10), and JAK2V617F positivity (IL2R) was demonstrated [44]. All of these findings represent factors independent from those used in the clinical Dynamic International Prognostic Scoring System (DIPSS).

## 2. MPNs and Oxidative Stress

Oxidative stress and inflammation both have possibly pathological actions in MPN. Moreover, oxidative stress is known to play a major role in hematological malignancies [45,46,47].

Several reports have demonstrated that oxidative stress and malondialdehyde (MDA) were increased in MPN subjects, which decreased in response to treatment, while the total level of antioxidants was lower with respect to healthy controls [48,49]. Oxidation protein products and S-nitrosylated proteins were also considerably increased in PV and ET subjects [50].

The influence of oxidative stress has been evaluated by testing total antioxidant capacity (TAC), total homocysteine (tHcy), cobalamin, holo-transcobalamin (HoloTC), and folate concentrations in MPN subjects, including patients with primary MF and with post-PV MF.

The analyzed subjects affected by myelofibrosis presented greater tHcy and higher values of ROS and lower TAC concentrations, confirming an altered oxidative status. Worsened MF forms were associated with lower TAC and HoloTC levels, the latter parameter being more frequently linked to Janus kinase-2 homozygosity [48].

**Figure 2:**
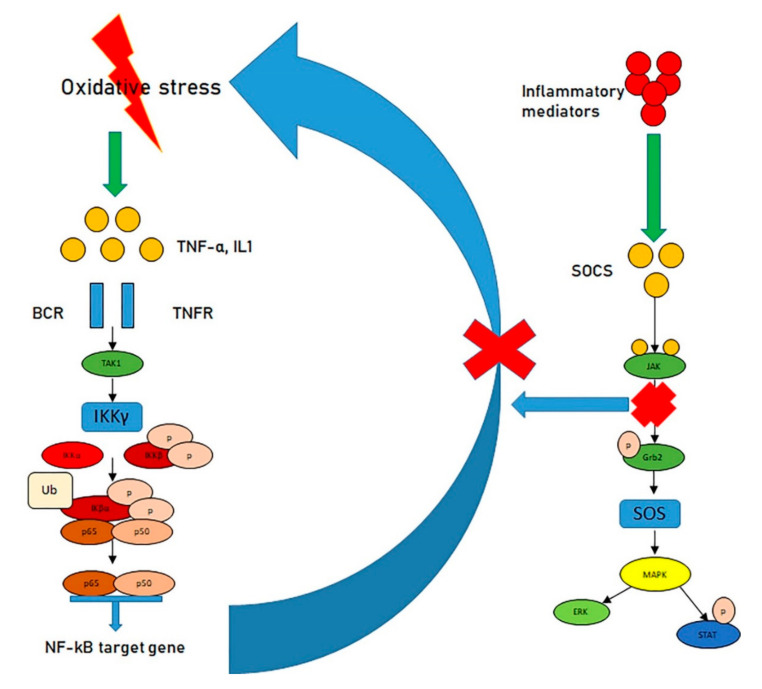
Oxidative stress, the NF-κB pathway, and the JAK–STAT pathway. Oxidative stress stimulates the NF-κB pathway, fostering a cycle in which oxidative stress generates ROS, which, in turn, produces more oxidative stress (**left**). However, inflammatory mediators stimulate the Suppressors of cytokine signaling (SOCS), in turn linking to JAKs, interrupting the JAK–STAT pathway (**right**) and, ultimately, blocking the inflammatory process.

Oxidative stress has been reported to stimulate the NF-κB pathway which, in turn, causes a self-perpetuating vicious circle in which oxidative stress generates ROS that in turn produces more oxidative stress [51,52,53]. To escape such conditions, the system employs the suppressors of cytokine signaling (SOCS), a group of proteins committed to producing negative feedback loops that are generally stimulated by inflammatory mediators [54,55]. The stimulated SOCS proteins link to JAKs and interrupt the JAK–STAT pathway, thus guaranteeing that the inflammatory process does not continue (Figure 2).

Unfortunately, in MPNs, this pathway is continuously stimulated, and SOCS control is removed. Furthermore, abnormal methylation of SOCS-coding DNA and malfunction of SOCS have also been demonstrated in MPNs [56,57].

ROS molecules are mainly generated by neutrophils, macrophages, and monocytes. In the case of MPNs, this is decisive as the MPN cells are clonal and have been demonstrated to generate disproportionate ROS both in vitro [58] and in vivo [59]. Augmented ROS generation has been reported in other types of tumors, and tumor cells have been shown to express catalase (the enzyme that metabolizes H_2_O_2_) in surplus and produce huge concentrations of H_2_O_2_. In this way, the tumor clone itself escapes the toxic action of H_2_O_2_ and destroys neighboring healthy cells (as ROS provoke programmed cell death in normal cells), thus supporting clonal proliferation [60,61,62,63,64,65,66,67,68].

In addition to the proliferation gain, the detrimental actions of ROS are also due to the subsequent oxidation of proteins, lipids, and dsDNA damage due to oxidation. In healthy cells, this damage will be quickly restored; however, a characteristic of most tumors is malfunctioning DNA repair. Moreover, the reaction to DNA injury is also altered by the negatively controlled p53 pathway in MPNs [69]. CHEK2 germline mutations, which have been correlated with ET and PV, account for an augmented risk of an MPN arising. Together with other substances, CHEK2 proteins are correlated with DNA injury, while the binding of TP53 (p53) and CHEK2 have been implicated in several tumor diseases [70,71,72,73,74,75,76].

It is also essential to consider that increased ROS has been recognized to control the actions of other signaling pathways implicated in tumor growth and programmed cell death, such as the phosphoinositide 3-kinase/protein kinase B (PI3K/PKB) and mitogen-activated protein kinase (MAPK) signaling pathways, through the oxidation of negative feedback loop controllers [77,78,79].

Furthermore, the activation of p38MAPK-mediated signaling in chronic inflammatory situations, such as in PV, alters proliferative activity and guides the option of cell destiny to programmed cell death or senescence [80,81]—cellular situations similar to the condition of fibrotic BM. In particular, disproportionate stimulation of the p38–MAPK cascade has been reported to be correlated with MF [82].

It is also likely that augmented endogenous ROS stimulates AKT/mTOR signaling in parallel with decreased antioxidant abilities and influences myeloproliferation in MPN.

Djikic et al. studied the impact of oxidative and nitrosative stresses on the stimulation of the AKT/mTOR signaling pathway in MPNs [83]. Gene expression of the antioxidants glutathione peroxidase 1 (GPx1) and superoxide dismutase 2 (SOD2) was increased in circulatory CD34+ cells, while levels of GPx1 and SOD1 enzymes were decreased in the erythrocytes of MPN. Plasma protein carbonyl concentrations and malonyl dialdehyde were increased in MPN. The whole antioxidant capacity in plasma and erythrocyte catalase activities was the most conspicuous in MF with *JAK2*V617F heterozygosity. The total nitrite/nitrate level was increased in the plasma of MF subjects, while inducible nitric oxide synthase (iNOS) and nitrotyrosine were usually augmented in the granulocytes of MPN subjects. Stimulation of AKT/mTOR signaling was the most relevant in MF, with hydrogen peroxide activating the mTOR pathway [83].

By employing transcriptional profiling, it has been demonstrated that several oxidative stress and antioxidative stress genes are considerably altered in MPNs. Among the twenty most up- and downregulated genes, *GPX8*, *ATOX1*, *PRDX2*, *PRDX6*, *PTGS1*, *SEPP1*, and *DEFB122* progressively increased from ET over PV to PMF, while there was a progressive decrease in *SIRT2*, *TTN*, *CYBA*, *UCP2*, and *AKR1B1* [84].

Finally, oxidative stress in the stem cell niche may also provoke hypoxia, at least in subjects with evolved MF. Hypoxia has been correlated with an augmented transcriptional action of hypoxia inducible factors (HIFs), including HIF-1alpha. Moreover, hypoxia is related to the increased expression of HIF-transcriptional targets, such as FoxO3, which is essential for preservation of the hematopoietic stem cell pool [85,86,87,88].

## 3. MPN Driver Mutations and Inflammation

In *BCR–ABL1*-negative MPNs, the driver mutations are found in *JAK2*, myeloproliferative leukemia virus proto-oncogene (*MPL*), and calreticulin (*CALR*). In each case, these mutations seem to accompany other alterations conditioning the onset of an inflammatory state.

The *JAK2* mutation has been correlated with more prominent redox alteration than presented by *JAK2*-negative MF subjects. It has also been reported that the *JAK2* mutation causes augmented ROS levels, mainly by altering catalase concentrations, a compound which is able to transform reactive H_2_O_2_ to H_2_O and O_2_ [58].

Transcriptomic analysis of HSCs from subjects with the *JAK2V617F* mutation displayed increased transcript levels of inflammatory markers such as TNFa, IFNa, and TGFb-related genes [89]. In the case of the STAT1 gene, transcript levels were increased in the *JAK2* mutant but not in *JAK2* WT cells nor NML cells.

Such findings are in agreement with other results from CD34+ cells of MPN subjects, where *JAK2V617F*—but not mutant *CALR*—caused an increase of IFNa-controlled genes [90]. A possible hypothesis regarding these results is that CALR-mutated MPN subjects require greater amounts of IFNa to attain an analogous molecular response [91], as confirmed by Czech et al., who reported higher doses of IFN were needed to achieve the same response in *CALR* mutant 32D cells than in *JAK2V617F* mutant 32D cells [90].

Stetka et al. reported that *JAK2V617F* stimulates an intrinsic IFNγ- and NF-κB correlated inflammatory procedure [92]. They demonstrated that cells with *JAK2V617F* strongly control concentrations of inflammatory cytokine-induced ROS, and when exposed to inflammatory cytokines, do not fully stimulate the ATM/p53/p21waf1 checkpoint and p38/JNK MAPK stress pathway signaling as well as repressing the expression of DNA single-strand-break repair genes while overexpressing dual-specificity phosphatase (DUSP) 1. These findings suggest that the *JAK2V617F*+ PV progenitors use DUSP1 activity as a defense against DNA injury, stimulating their growth in the inflammatory milieu and thus recognizing DUSP1 as a possible therapeutic target in PV [92].

Some authors have reported that loss of DNA methyltransferase 3 (Dnmt3) is capable of influencing *JAK2V617F* expression. In turn, *JAK2V617F* expression occurs at a higher rate in MPN progression [93]. To demonstrate their hypothesis, Jacquelin et al. employed clustered regularly interspaced short palindromic repeats (CRISPR) combined with CRISPR-associated protein 9 (Cas9) to inactivate Dnmt3. As a result, this blockade boosted the progression from *JAK2V617F* PV to MF [93].

The additional progression to MF in *JAK2V617F/Dnmt3a* double-mutant animals occurs through the activation of pro-inflammatory genes, which are able to increase TNFa through NF-κB pathways. Dnmt3a loss increased the release of pro-inflammatory mediators, especially when combined with oncogenes such as *FLT3-ITD* and *NRas*. Some alternations in gene expression that are able to activate TNFa signaling and pro-inflammatory gene expression as reported in the *JAK2V617F/Dnmt3a* double-mutant animals were also demonstrated in primary cells from MPN subjects with vs. without mutated *DNMT3A* [94].

The lack of TNFa in *JAK2*V617F-transduced BM cells entirely abolished the MPN phenotype in transplanted mice [8]. Moreover, it has been demonstrated that NF-κB signaling driven by TNFa is essential for inflammatory cytokine delivery [95]; however, this was abolished by *JAK2* blockade [96,97]. In a *JAK2V617F* transgenic animal model, the action of NF-κB/CDK6 in the control of inflammatory cytokines has been further highlighted [98]. These experiments demonstrated that CDK6 operates as an enhancer of the transcriptional action of NF-κB, where its blockade led to mitigation of the MPN phenotype through the diminution of NF-κB-dependent inflammation [99].

*CALR* is an endoplasmic reticulum (ER) chaperone acting in the regulation of protein folding1 and Ca^2+^ homeostasis [100]. CALR has also been demonstrated to be present in the cytoplasm and cell membrane, where it operates in the control of cellular stress responses [101]. Increased levels of CALR have been demonstrated to augment cell sensitivity to H_2_O_2_-provoked cytotoxicity [102], suggesting that CALR plays an essential role in programmed cell death induced by oxidative stress [103].

Although the mechanism of action due to *CALR* mutation has not yet been completely elucidated, its role in inflammation has been well explained by its oncogenic action, that is, working as an autocrine growth factor by joining to MPL. In fact, numerous researchers have verified that *CALR* mutation guides oncogenic transformation through MPL-dependent constitutive stimulation of JAK/STAT signaling [104]. The modification in the N-terminal domain between wild-type and *CALR* mutation, in terms of MPL-binding capacities, is due to structural changes provoked by the chemical properties of the mutant-specific C-terminal domain. Homodimerization of MPL is necessary for stimulating MPL-bound JAK2. Connecting mutant CALR to MPL does not provoke receptor dimerization; however, it may still structurally modify MPL similarly to when it is engaged by TPO [105].

The existing relationship between the driver mutation of MPNs and the family of S100A proteins has been considered to be of particular interest. This group has been demonstrated to be correlated with inflammation and myelopoiesis as well as being capable of causing myeloproliferation during chronic inflammation. Moreover, it has been stated that dimeric S100A8/9 might increase tumorigenesis by activating inflammatory cells and generating a pro-inflammatory BM milieu [106]. Furthermore, S100A4 has been reported to stimulate tumor cells to produce inflammatory cytokines, which trigger stromal cells to develop cancer-supportive properties [107].

S100A proteins have also been evaluated in MPN patients by Kovacic et al. They found augmented levels of S100A4, S100A6, S100A9, and S100A12 in CD34+ progenitor cells, especially in those with *JAK2V617F* and CALR mutations, with some differences between the MPN subgroups. In granulocytes, presence of *JAK2V617F* mutant allele was correlated with higher S100A levels and inflammatory status [108]. On the other hand, *CALR* mutation was associated with the reduced presence of S100A8, while the other S100 isotypes were not affected. The pro-inflammatory role of these proteins was further confirmed through their correlation to interleukin-8. However, the behavior of the single S100 was different, regardless of the MPN considered. At a molecular level, heterodimeric S100A8/9 was able to block the AKT pathway through Toll-like receptor 4 (TLR4) in granulocytes, while CALR mutation was capable of modifying the TLR4 response. Their experiment also determined that interfering with the receptor for advanced glycation end products (RAGE) did not influence the AKT pathway cascade. On the other hand, they noticed that the heterodimer S100A8/9 was capable of inhibiting the ERK 1/2 pathway, which is fundamental in PV [108].

Finally, in addition to the role of driving mutations, the presence of various polymorphisms could also influence the inflammatory state. To study the correlation between six polymorphisms in genes correlated to oxidative stress—specifically, the CAT-262 C > T, MnSOD Ala16Val, GPX1 Pro198Leu, GSTM1 and GSTT1 null genotypes, and GSTP1 Ile105Val—and the incidence of BCR–ABL-negative MPNs, 328 subjects having a known *JAK2 V617F*, MPL, and *CALR* mutation status were genotyped for these polymorphisms [109]. The CAT-262 C > T and GPX1 Pro198Leu polymorphisms were observed to be considerably less common, while the GSTP1 IleVal105 polymorphism was observed considerably more frequently in subjects with BCR–ABL-negative MPNs, independent of the molecular subtype (i.e., *JAK2*V617F or *CALR* mutated) [109].

These studies suggest the hypothesis that the modification of genes correlated to oxidative stress might control the risk of acquiring BCR–ABL-negative MPNs.

## 4. Inflammation and Thrombotic Complications in MPNs

Thrombosis is the principal reason for morbidity and mortality in MPNs, occurring in about 20–35% of subjects with PV, 15–30% with ET, and 10–15% with MF. The genesis of thrombosis in MPNs is multifactorial and derived from a composite interaction among blood cells, the endothelium, and the clotting system.

Apart from their actions on stem cells, the inflammatory condition and oxidative stress appear to be able to operate on diverse types of cells in the BM milieu, including stromal and endothelial cells. BM stromal cells have a relevant responsibility in the genesis of MPNs. The mesenchymal stromal GLI1-positive cells in the BM deeply cooperate with the megakaryocytes, and when the megakaryocytes were transcriptionally studied, an increased inflammatory signature was demonstrated concerning leukotrienes, arachidonic acid, and cytokines [110].

Endothelial cells are also very relevant agents of the inflammatory phenotype in MPNs [111,112].

Once more, the *JAK2*V617F mutation seems to be the main actor. This time, its role was demonstrated in endothelial cells derived from induced pluripotent stem cells (iPS). Guy et al. induced iPS from subjects with *JAK2*V617F-MPNs. STAT3- and AKT-augmented phosphorylation and the higher proliferation rate were indirect signals of a pro-inflammatory and pro-thrombotic status. Finally, in animals where mutant *JAK2V617F* expression was targeted in endothelial cells by a PDGFb-inducible cre^ERT2^ promoter, P-selectin was found to be increased in JAK2V617F-positive endothelial cells [113].

A different condition participating in thrombotic predisposition implicates endothelial dysfunction, which causes a pro-adhesive and pro-inflammatory surface promoting leukocyte and platelet binding and stimulation [114].

Inflammation and hemostasis are strictly correlated conditions, and the relationship between the immune system and coagulation, as a host protection tactic against pathogens, has recently been shown to lead to the conception of immune thrombosis [115]. Alteration of this system may provoke vascular disease and contribute to venous and arterial thrombosis in different pathologies [115]. Recent reports have underlined the influence of chronic inflammation on the MPN hypercoagulable condition as confirmed by the correlation between increased C-reactive protein and thrombosis [14].

Platelets are fundamental for hemostasis. However, they also participate in both inflammation and immunity, with additional activity in thrombo-inflammation. Their main function is in aggregation, which is carried out by adhesion molecules, creating intimate links with neutrophils and monocytes to form circulating platelet–leukocyte aggregates. These components of the blood have a positive feedback relationship with other fundamental vessel cells, such as of the endothelium. The activation cascade retrieves more mediators with a thrombo-inflammatory detrimental loop. In MPN patients, the abovementioned aggregate can activate a coagulation cascade with thrombosis being the direct consequence, which is typical in these subjects. The main problem is the relapsing course of this event if correct therapy is not promptly adopted. Interfering with the inflammatory status of these patients seems to be fundamental [116].

## 5. Targeting the Inflammatory Microenvironment

The opportunity for intervening in the inflammatory condition as a treatment in MPNs certainly appears fascinating; however, at present, the data in the literature do not appear to be conclusive.

Ruxolitinib, a JAK1/JAK2 tyrosine kinase inhibitor, is currently extensively employed in MPN-associated myelofibrosis therapy [117]. Ruxolitinib improves cytokine-dependent immunopathology in inflammatory conditions, and the reduction of cytokine signaling by ruxolitinib has also been reported in preclinical in vivo studies [118]. Administration of ruxolitinib caused a relevant reduction of increased IL-6 concentrations and regulation of augmented TNF-α concentration in animals bearing a *JAK2*-driven malignancy [119].

Bjørn et al. evaluated the effect of ruxolitinib in producing superoxide radicals and hydrogen peroxide by monocytes in blood samples from subjects with MF. They also examined its influence on RNA and DNA injury. The production of superoxide by monocytes was considerably decreased during ruxolitinib treatment, but no influence on the generation of hydrogen peroxide by monocytes or the global level of oxidatively altered RNA or DNA could be established. They concluded that ruxolitinib has little antioxidative capacity [120].

However, the reduction in erythrocytes after ruxolitinib administration could be due, in part, to the activation of eryptosis—suicidal erythrocyte death typified by cell shrinkage [121]—and cell membrane scrambling with phosphatidylserine translocation to the cell surface [122]. Cellular systems implicated in the accomplishment of eryptosis include oxidative stress [123], even though other reports have identified diverse mechanisms [124].

Other therapeutic approaches have been attempted. Recently, mounting proof has demonstrated that variation in the expression of microRNAs (miRNAs) can control essential procedures in HSPCs, such as growth, differentiation, inflammation, and tumor evolution [125,126,127,128].

The increase in miR-382-5p levels in MF CD34+ cells has also been recently observed. To disclose the action of miR-382-5p in the genesis of MF, a gene expression profiling study of CD34+ cells overexpressing miR-382-5p was conducted. SOD2 was amongst the repressed genes, which is a predicted target of miR-382-5p. miR-382-5p increase in CD34+ cells provokes a reduction in SOD2 expression, causing ROS increase and oxidative DNA injury. Moreover, the results suggested that silencing of miR-382-5p in MF CD34+ cells re-establishes SOD2 activity, causes ROS clearance, and decreases DNA oxidation. As the pro-inflammatory cytokine TGF-b1 plays a central role in MF genesis, one study evaluated the action of TGF-b1 on ROS and miR-382-5p concentrations. Their results demonstrated that TGF-b1 administration increases miR-382-5p expression and decreases SOD2 action, causing an increase in ROS.

A method to regulate inflammation in MPNs implicates the employment of galunisertib, a small molecule antagonist of the tyrosine kinase transforming growth factor-beta (TGF-b) receptor type 1 (TGFBR1) with possible antineoplastic action. After dispensation, galunisertib exclusively connects to the kinase domain of TGFBR1, thus avoiding the stimulation of TGF-b-mediated signaling pathways. Blockage of TGF-b1 signaling in MF CD34+ cells by galunisertib considerably decreased miR-382-5p expression and ROS increase while resulting in re-established SOD2 activity. Thus, it is conceivable that alterations in the TGF-b1/miR-382-5p/SOD2 axis in MF cells is due to ROS accumulation that, in turn, may provoke increased oxidative stress and inflammation. Galunisertib might be an efficacious drug in decreasing the altered oxidative stress state caused by TGF-b1 in MF subjects [129].

Attempts to intervene in the systems that control the production of cytokines and oxidative stress could also prove efficacious. Components of the bromodomain and extra-terminal (BET) group of proteins operate as “readers” of histone modification marks by interacting with acetylated lysine residues on histone tails in addition to control genes that have been implicated in inflammation and tumor, such as *BCL-2*, *MYC*, and *NF-*κB [130,131]. The NF-κB pathway downstream to BET has been demonstrated to be stimulated in MF through numerous inflammatory cytokines [132].

In a preclinical report employing two different MF animal models, the administration of a BET inhibitor caused a decrease in cytokine delivery, spleen size, and BM fibrosis; these negative changes were attenuated by the dispensation of ruxolitinib [133]. These findings suggest that substances that decrease genes influencing NF-κB might be efficacious in curing MF. CPI-610, a BET inhibitor, is presently being evaluated for the treatment of MF subjects, alone or in combination with ruxolitinib (NCT02158858).

In the attempt to reduce inflammation and oxidative stress, statins may also be extremely effective (though always in a combinatorial treatment), as they have been reported to inhibit MPN cell proliferation and augment the action of JAK2 inhibition [3,10].

Finally, transplanting mice with a mixture of *JAK2*-mutated and *JAK2* wild-type HSCs generated a PV phenotype, the onset of which could be blocked by the dispensation of antioxidants such as N-acetylcysteine (NAC). In an in vivo experimental model, NAC administration decreased the level of DNA injury as demonstrated by a decrease in 8-oxoguanines and double-stranded DNA breaks [58].

Hence, the employment of antioxidant substances could be advantageous in the treatment of MPNs.

## 6. Conclusions

The above-cited papers have confirmed that MPNs are diseases which are sensitive to ROS overload. The pro-inflammatory status induced by oxidative stress augments the risk of malignant transformation. Many aspects concerning the relationship between chronic myeloproliferative diseases, genetic alterations, and inflammation remain to be clarified.

In conclusion, MPNs are an assorted set of acquired clonal diseases where an anomalous hematopoietic stem cell alters myeloid progenitors, causing the increased generation of one or more forms of myeloid cells. In recent years, several studies have suggested that chronic inflammation may play an essential role in their onset and progression with the involvement of genes involved in inflammasome activation. Inflammatory cytokines are also capable of influencing disease onset as well as its evolution and prognosis. Oxidative stress, by promoting inflammation, has been speculated as being involved in the onset of MPNs. In fact, ROS trigger inflammatory pathways, causing a self-perpetuating vicious circle. Finally, the BCR–ABL1-negative MPN driver mutations—that is, in *JAK2*, *MPL*, and *CALR* genes—seem to be able to accompany other alterations, conditioning the onset of an inflammatory state. This altered state is also sustained by platelets which participate in inflammation and immunity. In particular, thrombo-inflammation facilitates an additional inflammatory pathway in MPN. The opportunity for intervening in the inflammatory condition as a potential treatment for MPNs certainly appears fascinating; however, at present, the data in the literature do not appear to be conclusive. The employment of antioxidant substances could be advantageous in the treatment of MPNs. Ruxolitinib, a JAK1/JAK2 tyrosine kinase inhibitor, is currently being extensively employed in MPN-associated myelofibrosis therapy, but it has little antioxidative capacity. MicroRNAs, statins, and galunisertib have also been studied as therapeutic agents, though no lasting remedial results have been obtained thus far.

Recognizing the genetic and external elements that participate in the myelofibrotic evolution of MPNs is crucial for early detection as well as to initiate therapies that can inhibit or reverse disease development in MPN patients.

In the future, the inclusion of inflammation markers in studies on MPNs may provide important insights into the mechanisms of action of these diseases and help in defining new combination therapies.

## Figures and Tables

**Table 1:** Overall one-to-one comparison of cytokine/chemokine balance between: (i) MF and ET; (ii) MF and PV; (iii) ET and PV; and (iv) ET and controls. Each of the four comparisons highlights the cytokines/chemokines displaying higher concentration in one condition with respect to the other (i.e., to which the comparison is conducted).

**Myelofibrosis (MF)**	**Essential Thrombocythemia (ET)**
IFN-γ IL-17a IL-12p70	
**Myelofibrosis (MF)**	**Polycythemia Vera (PV)**
TNF-α GM-CSF IL-4 IL-12p70	
**Essential** **Thrombocythemia (ET)**	**Polycythemia Vera (PV)**
RANTES MIP-1	
**Essential Thrombocythemia (ET)**	**Controls**
RANTES	
